# Physiological Stress in Rescued Wild Koalas Are Influenced by Habitat Demographics, Environmental Stressors, and Clinical Intervention

**DOI:** 10.3389/fendo.2019.00018

**Published:** 2019-01-29

**Authors:** Edward Narayan, Tayla Vanderneut

**Affiliations:** ^1^School of Science and Health, Western Sydney University, Penrith, NSW, Australia; ^2^School of Animal and Veterinary Sciences, Charles Sturt University, Wagga Wagga, NSW, Australia

**Keywords:** koala, rescue, rehabilitation, stress, environmental trauma, disease

## Abstract

Koalas are rescued from the wild often with incidence of burns from bushfire, injury from animal attacks, vehicle collision, and diseases. Exposure to environmental stressors (trauma and disease) could generate physiological stress and potentially impact the outcomes of clinical management intervention and rehabilitation of rescued wild koalas. It is important to quantify the stress physiology of wild koalas upon registering into clinical care. This study demonstrates the first report of physiological stress assessment in rescued wild koalas (*n* = 22) to determine the potential influences of habitat-specific demographics, stressor category, and clinical diagnosis. Fecal samples were collected from the koalas at rescue and routinely during hospitalization to provide a longitudinal assessment of the koala's stress response throughout clinical care. Fecal glucocorticoid metabolites (FCM) enzyme-immunoassay was used to index physiological stress non-invasively. Koalas were admitted with exposure to various categories of environmental trauma such as vehicle collision, dog attack, burns from forest fire (this also related to conditions such as copious drinking and flat demeanor), and other injury. The main disease diagnosed was chlamydial infections. In terms of environmental interactions, it was found that habitat-specific demographics, location where the rescued koala was found, especially the rural-urban fringe, influenced FCM levels. Furthermore, there was significant interaction between location, stressor category, and clinical diagnosis for mean FCM levels. However, these factors were not predictive of the clinical outcome (euthanized or released). Overall, the results provide invaluable insights into how wild koalas respond physiologically to environmental trauma and disease and how methods of care, husbandry, and treatment can be used to further reduce the impacts of stress with the ultimate aim of increasing the rehabilitation and future release of rescued koalas to revive the declining mainland populations.

## Introduction

Global biodiversity is in rapid decline with an increase in human use of Earth's natural resources (1). Australia is home to some of the world's most distinctive and unique fauna with 80 percent of its terrestrial mammalian species being endemic (1). However, worldwide mammalian biodiversity is showing rapid declines largely due to factors such as habitat degradation and hunting (1). It is estimated that over 50 percent of all mammal species extinctions worldwide over the past 200 years are from Australia (2). Since 1788, 28 Australian endemic land mammals have become extinct and this rate is increasing (1). These figures make Australia the worst record for mammal conservation with rates of extinction exceeding that of any continent (2, 3). There are a multitude of both environmental factors and species attributes being recognized as causations of this species decline (3). These are inclusive of anthropogenic induced environmental changes (4), shelter and foraging habitat, regional productivity, fecundity, longevity and phylogeny (3). Further factors include the introduction of predators such as cats and foxes as well as incidence of infectious diseases (3). The International Union for Conservation of Nature (IUCN) now lists 56 Australian land mammals as threatened and an additional 52 as near-threatened (1). One these threatened species is the koala (*Phascolarctos cinereus*), being recognized as threatened under both Commonwealth and State legislation (5).

Koala mortality is of increasing concern with multiple environmental and anthropogenic factors attributing to this species decline (6). Disease has been considered as one of the prevalent causes of losses (7). Both retrovirus and trypanosomes are some of the pathogens affecting koala losses however the most recognized is the incidence of Chlamydia (6). A review of historical records has recognized chlamydiosis symptoms to be present in cases as early as the 1800s (6). Symptoms associated with the disease are inclusive of kerato-conjunctivitis, pneumonia, urinary tract infections, and genital tract infections, especially in female koalas (6). These can cause adverse effects such as infertility in some koala populations (6). The spread of the disease is also Australia wide in both captive and wild populations, with little indication suggesting that it is location specific (6). Currently the diagnosis of chlamydia requires intense clinical examination including PCR detection and ultrasonography (6, 8). In general, the disease is usually presumed in koalas experiencing some of the symptoms such as sore eyes, chest infections, and “wet bottom” or “dirty tail” (6). In a wildlife hospital or clinical setting, the infection is treated through the use of antimicrobial drugs but the results thus far are mixed (6). There has been progress in the development of a chlamydial vaccine to control the disease in koala populations (6).

Further associated factors of mortality and injury to the wild koala is vehicle collision, bushfire and dog attacks (9, 10). Vehicle collision are of particular concern in heavily urbanized environments where there are small fragmented koala populations (9). In particular localities, such as Phillip Island in Victoria, vehicle collisions make up for 60% of the mortality for koala populations (9). The incidence of bushfire also threatens koala population survival causing burns and respiratory issues to individuals (10). Like the trends of road mortality, bushfire frequency is heightened in areas of habitat fragmentation (10).

The hypothalamo-pituitary adrenal (HPA) axis is active during stress, which causes release of corticotropin releasing-hormone (CRH), which travels through the hypophyseal portal system to release adrenocorticotropic hormone (ACTH) from the anterior pituitary and into the blood stream (11). ACTH then acts to release glucocorticoid (GC) steroid hormone from the cortex of the adrenal gland. GCs can either be in the form of cortisol or corticosterone and dependant on the species, either cortisol or corticosterone, or even both, are produced. Cortisol is the major GC in mammals (eutherian and metatherian species) while corticosterone is the major GC in fish, amphibians, reptiles, and birds. The effects of GCs can last from several minutes to hours. Depending on amount by which GCs are elevated can provide an insight into the severity of the stressor and how an animal reacts to it (12, 13). In koalas, cortisol has been identified as the major circulating GC (14), however both cortisol and corticosterone metabolites have been measured in excreta (15, 16). Levels of FCMs in adult healthy male and female koalas have been reported earlier in response to an ACTH stimulation test as follows; Pre-ACTH challenge; males (7.1 ± 1.29 ng/g dry feces, *n* = 6) and females (3.9 ± 0.51 ng/g dry feces, *n* = 18). Mean fecal cortisol metabolite concentrations in the males and females after the ACTH challenge were as follows: Males (8.9 ± 0.80 ng/g dry feces, *n* = 19) and females (6.7 ± 0.47 ng/g dry feces, *n* = 12).

The types of stressors and their duration can provoke an array of neuroendocrine responses and immunity capabilities of an individual (17). There is a proposed link between environmental factors affecting koala population declines such as the modification of landscapes and disease incidences, and the effect of physiological stress on immune capabilities (18). It is recognized that there is an influence of stress on disease susceptibility in wildlife species (18). Prolonged stressors or chronic stressors, result in reductions of basic immune processes (19). Short term stressors (acute stressors) however generally enhance immune responses (19). Baseline stress then describes the absolute basal levels of stress hormone secretion experienced by the individual in a state where there are no posed threats (20, 21). It is recognized that baseline GC levels have the ability to change as the organism encounters environmental fluctuations and therefore stress causes elevation of cortisol secretion (21). In wild koala populations there is no knowledge of cortisol levels in rescued koalas.

An understanding of the relationships between stress, incidence of disease and trauma and clinical outcomes is key for conservation management of wildlife populations (22). The measurement of GCs is key into investigating these relationships as they are able to indicate the stress response and physiological resilience of the animal (22). The use of non-invasive techniques such as fecal GC metabolite measurements is a significant tool to measure the stress responses whilst not increasing stress responses through invasive interactions (i.e., blood collection) (15). Being a folivore with a natural diet consisting of *Eucalyptus* spp., which is extremely high in fibers, the koala requires a long gut system to be able to digest these products (23). In general, diets that are higher in fiber will cause a delay in GC release and gut transit time (24). It is currently approximated that digestion and GC transition to feces will take an average of at least 213 h (23). In koalas, fecal based hormone monitoring technique is highly suitable due to their long gut system and therefore a lengthy excretory lag-time of over 9 days. Therefore, the first fecal sample collected at rescue provides a window into quantifying the physiological stress responses of koalas to environmental stressors (15).

The success of wildlife rehabilitation is based on successful treatment as well as long term survival and ultimate release of the patient koalas (25). Fertility is also a leading driver in the success of rehabilitation (26). In general, there is a greater need for research in the rehabilitation process (26). Currently the success of chlamydial treatments such as topical ointment and antibiotics is lacking with high failure rates of recovery (26). Whilst there has been exploration surrounding infection treatments in clinical settings, there is no research that investigates if prior life experiences have impact on an animal's recovery and outcomes. In this study, we attempt to find out the effects of environmental stressors on the outcome of koalas in a clinical setting.

The measurement of fecal glucocorticoid metabolites and the koalas long gut system therefore allows us to have an understanding of the stress experienced by koalas several days before arrival to the clinic and also gives indication as to whether absolute baseline stress levels could affect clinical outcomes. It is hypothesized that those rescued koalas admitted to the veterinary clinic experiencing prior heightened stress levels (e.g., burn victims from bush fire) will have lowered success to recovery in the clinic and will be mainly euthanized.

## Materials and Methods

### Study Koalas

This study was done through formal approval by the Charles Sturt University ACEC Committee (Protocol number: A16044). Koala health data was collected in partnership with Adelaide Koala and Wildlife Hospital (AKWH), South Australia. The hospital is dedicated to the emergency treatment, rehabilitation of injured or orphaned native wildlife. During the koala's admittance in clinic, they were housed individually in large cages and provided with fresh water and various assortments of Eucalyptus species. Sampled koalas were those in care at AKWH during the sampling period of 2015–2016.

#### Health Data, Habitat Demographics, and Stressor Categories

Health data provided was inclusive of hospital records for the koalas with matched fecal sampling done (*n* = 22). Hospital records contained details of health checks, age, sex, weight, stressor categories, treatments, and outcomes.

Using the AKWH records that were provided, a health summary was created for each koala which detailed their basic information (age, sex, location found, etc.) and then what treatment was used, treatments administered, how long they were in hospital for, and what their outcome was. Stressor category, location, and clinical outcomes were all categorized to allow for statistical analysis.

Habitat was categorized using Google Maps to identify the habitat demographics where the koala was found by rescuers. The habitat demographics categories included; “National Park” which indicated that the koala was picked up from within a national park, “Rural” which indicated an area that was sparsely populated and mainly included large lots of grass lands and open areas, “Semi-Urban” which was a location that was moderately populated and situated near or fringed by parkland, forest, or open grasslands, and “Urban” which were areas that is densely populated and a distance from any forests or parklands.

Stressor categories were as follows; Healthy koalas were identified as with good body condition score of >4.0 (27) and no physical signs of disease. Suspected infection cases showed physical signs such as red/swollen/sore eyes/conjunctiva, discharge, red cloaca, wet bottom, swollen genital however tested negative for chlamydia (PCR testing return negative). Injury included physical injuries sustained from any physical trauma apart from dog-attack or vehicle collision. Burn victims were koalas that were rescued from bush fire impact. Dehydrated patient identified as a koala that was found to be drinking an abnormal quantity of water for an abnormal length of time (e.g., some rescued koalas recorded drinking for over 40 min). Flat demeanor was noted when a rescued koala was found in a state of not exhibiting normal behaviors, seemed slow and depressed or was not responding to external stimulus appropriately.

Diagnosis was determined through veterinary testing and examinations. For example, a koala that had vehicle collision was found to have multiple fractures so this is what it was ultimately treated for. Another koala may have been found on the ground but ended up being treated for an infected pouch, so infected pouch was its diagnosis. Due to the nature of chlamydia and its intermittent shedding, the PCR tested negative or positive was used as a diagnosis as a –ve or +ve result could influence the FCM levels. Other common diagnosis included renal failure, arthritis (inability to climb), diabetes and respiratory illness.

### Fecal Sample Collection

Fecal samples were collected from 22 koala patients admitted to the AKWH from the period of 2015–2016. During routine cage cleaning, 1–5 fresh pellets were collected from each koala daily at the same time period in the morning to avoid potential influence of circadian rhythms on FCMs. Sample size (days) ranged from *n* = 2 days−36 days depending on the length of time that each koala stayed in the clinic. Fresh pellets were initially identified by intensity of smell, mucous covering and lack of dehydration. Samples were placed into Ziplock© bags and labeled with the animal's name, date, identification number, and time of sample collection. Samples were stored at −20°C until they were sent on ice to the laboratory *via* overnight freight. Upon delivery, the fresh fecal samples were immediately frozen to minimize effects of sample age on FCM levels. All samples were analyzed within 1 month of collection.

### Sample Preparation

Frozen fecal samples were dehydrated in a freeze dryer for a 24 h period (or until completely dried). Once dry, samples were ground into a fine powder up using a mortar and pestle. Each mortar and pestle was cleaned using 10% ethanol between samples. The ground up powder was sifted through a find mesh strainer to remove all course particles. A 0.2 grams (g) +/− 0.001 g sample of sifted product was weighed out into a labeled test tube and then stored in a −20°C freezer.

### Fecal Cortisol Metabolite Extraction

Samples were removed from the −20°C freezer and 2 milliliters (mL) of 90% ethanol solution was added to the test tube. Tubes were vortexed at medium-high speed on an Eppendorf mini-spin centrifuge for a minimum of 30 s to thoroughly mix the solution. Tubes were then placed into a +80°C water bath for 10 min to allow hormones to dissolve in the solution. Whilst in the bath, tubes were gently shaken to ensure feces stayed submerged in ethanol and did not spill over the top of the tube. After 10 min, the contents of the tube were poured into an Eppendorf tube, closed and then centrifuged at 10,000 RPM for 5 min until the liquid residue separated from the hormones dissolved in ethanol. Following this, 0.6 mL was aliquoted into a new, clean, and labeled Eppendorf tube. Tubes were left open and stored in a laminar flow chamber for a minimum of 24 h until the ethanol has evaporated and the tube was completely dry. Once tubes were completely dried, 1 mL of assay buffer (39 mM NaH_2_PO_4_, 15 mM NaCl and 0.1% bovine albumin, pH 7.0) was added to the tube. Clean pipette tips were used to scrape off as much of the residue as possible. Tubes were vortexed at medium-high speed on an Eppendorf mini-spin centrifuge for a minimum of 30 s. Following this, they were centrifuged at 10,000 RPM for 10 min. After centrifugation, 850 microliters (μL) of supernatant was pipetted into a clean labeled Eppendorf tube avoiding any of the solid section of the solution when pipetting. If the sample appeared to still be cloudy, tubes were re-centrifuged for 10 min and then pipetted again into a new tube. Samples were then stored in a −20°C freezer until ready for use.

### Hormone Analysis

Validation of the fecal cortisol metabolites (FCM) extraction method is described in (15) and follows the previously described extraction protocols of (28–30). FCM concentrations were determined using a polyclonal anti-cortisol antiserum (R4866) diluted to 1:15,000, horseradish peroxidase (HRP) conjugated cortisol 1: 80,000 and cortisol standards (1.56–400 pg well^−1^). Sample extracts were then assayed in duplicate on Nunc Maxisorp™ plates (96 wells). Plates were coated with appropriately diluted cortisol antibody and left to stand and incubate for a minimum of 12 h in a fridge at 4°C. The plates were then washed using an automated plate washer (ELx50, BioTek™) with phosphate-buffered saline containing 0.05% Tween 20. The dilution factor for the FCMs in koala fecal extracts where based on the concentration of pooled samples that resulted in 50% binding on the parallelism curve [see (15)].

For each assay, 50 μL of cortisol standard, control, and diluted fecal extract was added to each well-based on the plate map, immediately following 50 μL of HRP was added. Plates were covered and incubated at room temperature for exactly 2 h. After 2 h of incubation, plates were washed and 50 μL of substrate buffer (0.01% tetramethylbenzidine and 0.004% H_2_O_2_ in 0.1 M acetate citrate buffer, pH 6) was added to each well to generate a color change. Color reaction was halted after 15 min using 50 μL of stop solution (0.5 molL^−1^ H_2_SO_4_). To quantify the concentration of FCM in each sample the plates were read at 450 nm (with reference to 630 nm) on an ELx800 (BioTek™) microplate reader.

### Statistical Analysis

Data was statistically analyzed using SYSTAT software version 13.0. All FCM data was first log transformed to meet the assumptions of normality. Graphs were plotted in GraphPad Prism software. All FCM data points (from rescue to end point of clinical recuperation) for each koala were used to calculate mean levels that provided absolute baseline levels of FCMs for each koala. A GLMM ANOVA was used to compare level of significant difference between mean FCM (variable) and factors included (sex, koala ID, length of stay, stressor category, habitat location, diagnosis, and clinical outcome). *Post-hoc* comparison for interaction between habitat location, stressor category, and clinical diagnosis as determinants of mean FCM levels was done using Dunn's multiple comparison test. *P* < 0.05 was used as the level of significance.

## Results

### Mean FCM Levels Relative to Koala Habitat Demographics

GLMM Analysis of Variance results showed that mean FCM levels were significantly different between individual koalas (*F* = 26.33, df = 11, 220; *p* < 0.001). There was no significant difference in mean FCM levels between male and females, however the length of stay in the hospital was significant (*p* < 0.05).

Stressors experienced in rural localities included; vehicle collision, dog attacks, flat demeanor (associated with bushfire) and having a wet “dirty” bottom. Vehicle collision was the leading stressor in rural localities making up 33% of cases. All other stressors in rural habitats recorded occurrence of 17%.

Stressors experienced in semi-urban (rural-urban fringe) localities included; continuous drinking, eye discharge and flat demeanor, all of which were equally high occurrence at 27%.

Individuals in urban habitats experienced multiple stressors including; continuous drinking, dog attacks, eye discharge, flat demeanor and vehicle collision. Eye discharge had the highest occurrence (29%) followed by vehicle collisions and flat demeanor both at equal occurrence of 21%.

### Analysis of Factors and Interactions With Mean FCM Levels

#### Mean Fecal Cortisol Metabolites (FCM) Levels by Locations

**Figure 1:**
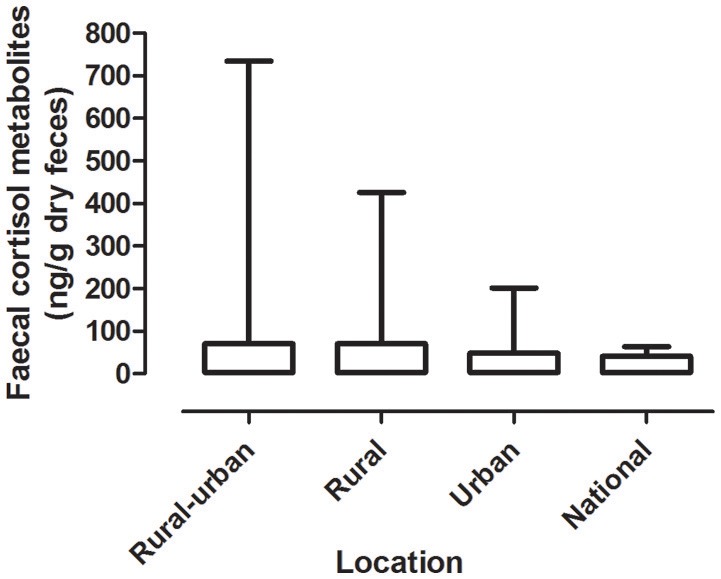
Shows the mean + range of fecal cortisol metabolites (FCM) levels in koalas distributed by locations.

Mean levels of FCM were not significantly different between locations (*F* = 1.31, df = 3, 167, *p* = 0.27; Figure 1). The highest mean FCM levels were present in koalas found at rural-urban fringe or semi-urban localities followed by rural and urban locations (Table 1). Koalas rescued from national parks had lowest mean FCM levels (Table 1). *Post-hoc* comparisons showed significant difference (*p* < 0.05) between all location comparisons, except for comparisons between urban vs. national park and rural vs. rural-urban fringe (*p* > 0.05 for all comparisons; Table 1).

**Table 1:** Shows the descriptive statistics and *post-hoc* comparisons of fecal cortisol metabolites (FCM) levels in koalas distributed by location.

**Category**			**Fecal cortisol metabolites (ng/g dry weight)**					**Statistical comparisons**	
**Number**	**Location**	**Sample size**	**Min**	**Max**	**Median**	**Mean**	**S.E.M**	***post-hoc*** **comparison (c.q.)**	**Significant (*p* < 0.05)**
1	Urban	77	3.26	202	24	48.34	5.492	I c.f.2	yes
2	Rural-urban fringe	47	13.42	734.6	38.39	69.87	15.69	I c.f. 3	yes
3	Rural	27	2	426.4	31	68.48	17.32	I c.f.4	no
4	National Park	17	15.82	64.22	36.85	39.74	3.395	2. c.f.3	no
								2 c.f.4	yes
								3. c.f.4	yes

#### FCM Levels by Stressor Category

**Figure 2:**
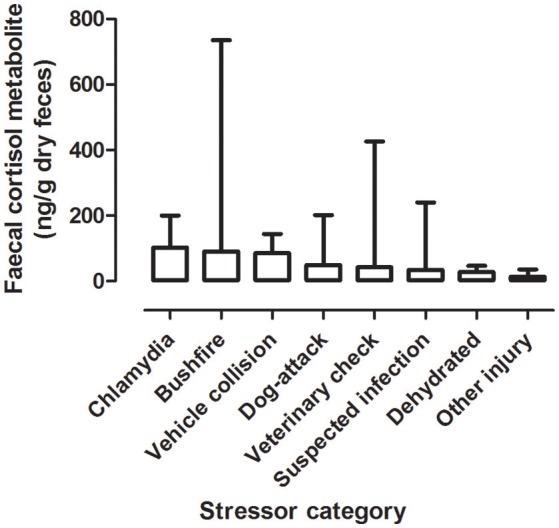
Shows the mean + range of fecal cortisol metabolites (FCM) levels in koalas distributed by stressor categories.

There was a significant difference between mean FCM levels for the different stressor categories (*F* = 5.33; df = 7, 240; *p* < 0.001; Figure 2). FCM levels were highest for koalas with chlamydia), followed by koalas impacted by bushfire (including burns and flat demeanor), vehicle collision, dog-attack, veterinary check, suspected infection, dehydration, and other injury (Table 2). *Post-hoc* comparisons showed that only level of significant difference in FCM levels were between bushfire vs. veterinary check and bushfire vs. other injury (Table 2).

**Table 2:** Shows the descriptive statistics and *post-hoc* comparisons of fecal cortisol metabolites (FCM) levels in koalas distributed by stressor category.

**Category**			**Fecal cortisol metabolites (ng/g dry weight)**					**Statistical Comparisons**	
**Number**	**Stressors**	**Sample Size**	**Min**	**Max**	**Median**	**Mean**	**S.E.M**.	***post-hoc*** **comparison (c.f.)**	**Significant (*p* < 0.05)**
1	Other injury	20	6	36	9.5	13.55	2.067	4 c.f. 7 and I c.f. 7	yes
2	Dehydrated	7	14	47	26	27.71	4.96	All other pairwise comparisons	no
3	Suspected infection	14	7	240	16	34.14	16.13		
4	Veterinary Check	120	3	426	24.5	42.69	4.837		
5	Dog-attack	12	14	202	30.5	49.17	15.36		
6	Vehicle collision	10	22	144	83	85.5	15.2		
6	Bushfire	53	2	735	75	91.09	13.55		
7	Chlamydia	5	54	200	76	102.4	26.62		

*Data for healthy koalas were referenced from earlier published work (15)*.

#### Mean FCM Levels by Diagnosis

**Figure 3:**
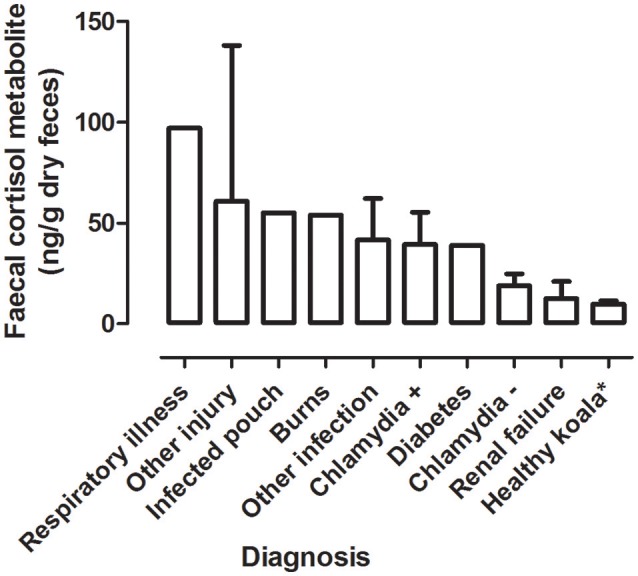
Shows the mean + range of fecal cortisol metabolites (FCM) levels in koalas distributed by diagnosis. *Healthy koala data was referenced from published study (15).

There was a significant difference between the mean FCM levels for diagnosis (*F* = 3.96; df = 5, 50; *p* = 0.0046; Figure 3). Koalas that were diagnosed with respiratory illness had the highest mean FCM, followed by respiratory illness, other injury, infected pouch, burns, other infection, Chlamydia +, diabetes, Chlamydia –, renal failure, healthy koala (Table 3). *Post-hoc* comparison showed level of significant difference only between comparison of healthy koala vs. other injury. A caveat here is low sample sizes for some of the diagnosis (see Table 1). Thus, categories with *n* = 1 sample size were excluded from the statistical analysis.

**Table 3:** Shows the descriptive statistics and *post-hoc* comparisons of fecal cortisol metabolites (FCM) levels in koalas distributed by diagnosis.

**Category**			**Fecal cortisol metabolites (ng/g dry weight)**					**Statistical comparisons**	
**Number**	**Diagnosis**	**Sample Size**	**Min**	**Max**	**Median**	**Mean**	**S.E.M**	***post-hoc*** **comparison (c.f.)**	**Significant (*p* < 0.05)**
1	Chlamydia–	3	8	8		28	5.859	All pairwise comparisons except,	no
2	Chlamydia+	8	5	5		146	16.02	4 c.f. 7	yes
3	Other infection	5	9	9		118	20.59		
4	Other injury	4	21	21		138	26.22		
5	Burns	1	54	54		54			
6	Renal failure	2	4	4		21	8.5		
7	Healthy koala1^*^	29	2.153	2.153		46.44	1.681		
8	Infected pouch	1	55	55		55			
9	Respiratory illness	1	97	97		97			
10	Diabetes	1	39	39		39			

* *Healthy koala data was referenced from published study (15)*.

In all cases of diagnosis for renal failure, arthritis (inability to climb) and diabetes the outcome was euthanasia. For koalas with diagnosis of burns, heat stress and respiratory illness, all cases ended with release. Diagnosis of chlamydia, other infections and injuries had cases of both release and euthanasia outcomes.

#### Mean FCM Levels by Multiple Factors

**Figure 4:**
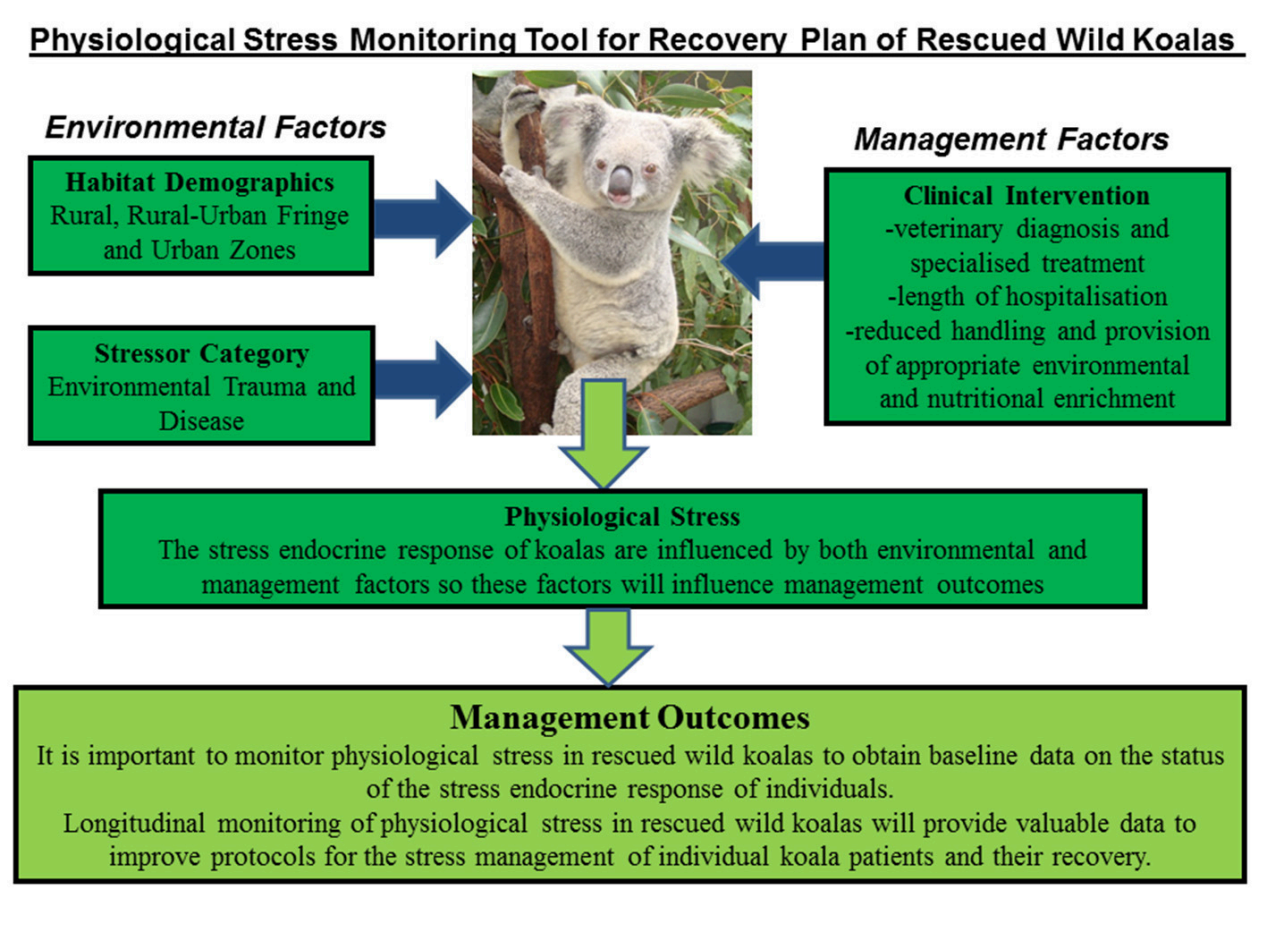
Shows the conceptual diagram summarizing the main findings of this study. That is, physiological stress in rescued wild koalas has the influence of both environmental factors (pre-rescue) and management factors (post-rescue). The pre-rescue factors include anthropogenic induced environmental stressors that generate physiological stress in wild koalas such as habitat fragmentation, forest fires, vehicle collision, dog-attacks etc. The habitat-specific factors, especially rural-urban fringe zone create ecological problems for koalas associated with accessing habitat and food sources. The post-rescue factors are mainly associated with the clinical management of koalas which include their veterinary care and diagnosis, length of stay, treatment, and rehabilitation. It is important to carefully assess physiological stress in wild rescued koalas in order to obtain real-time data on their physiological status at the point of rescue and to apply fecal glucocorticoid monitoring in clinical care to better understand their physiological response to human interventions. The application of non-invasive hormone monitoring can assist us to better manage and reduce stress for koalas under human care.

Significant interaction (^*^) was found between location, stressor, diagnosis, and outcome as predictors of FCMs levels in the koala patients (Figure 4). The test results were as follows:

$$\begin{array}{l} \;{\text{Location}}^{*}\text{diagnosis}(F=28.87,p=0.00)\\ \;{\text{Location}}^{*}{\text{stressor}}^{*}\text{diagnosis}(F=14.89,p=0.00)\\ \;{\text{Location}}^{*}{\text{stressor}}^{*}\text{outcome}(F=3.16,p=0.044)\\ {\text{Location}}^{*}{\text{Stressor}}^{*}{\text{diagnosis}}^{*}\text{outcome}(F=25.09,p=0.00). \end{array}$$

## Discussion

This study has provided new knowledge on the physiological stress responses of rescued wild koalas in relation to their habitat demographics, stressor category, and clinical intervention. The results showed that all of these factors interacted to influence levels of physiological stress (indexed using fecal GC metabolites) in the rescued koalas (Figure 4). Therefore, the clinical outcome (release or euthanasia) can be influenced by both the pre-rescue conditions as well as the clinical environment that is provided to the koalas in care.

Koalas that were rescued from rural-urban fringe locations had the highest FCM values while those in urban had the lowest (excluding national park and unknown). In rural locations, interestingly road collision was a leading stressor in 33% of rural cases. A study by Griffith et al. (31) on trends of koala admission to wildlife hospitals found that male koalas to have increased risks of vehicle accidents during the summer period where tourism was high. Griffith et al. (31) further found that vehicle accidents coincided with periods of land clearance with those koalas experiencing these anthropogenic induced threats to be more likely to be admitted to the wildlife hospital. Furthermore, (32) in their study compared major and minor roads and found the incidence of road mortality to be much greater on minor (rural) roads. It was also found that minor roads caused greater habitat destruction than the major roads of urban environments (32). Of the koalas found in rural locations (*n* = 6), four of these ended with a final outcome of euthanasia. Koalas in rural locations will experience less exposure to human activities compared to those in urban environments (33). However, koalas in an urban environment are found to be more resourceful, using all trees in the area, being able to better exploit patchy areas and increased ability to find mates in fragmented landscapes due to a life history of adaption to these experiences (33).

In both semi-urban and urban environments, eye discharge was at the highest occurrence. In semi-urban environments, other factors such as excessive drinking and sitting on the ground had equal high occurrence. Eye discharge was generally diagnosed as kerato-conjuntivitis, which is a leading symptom of Chlamydia (34). Red cloaca, eye discharge, wet bottom, and swollen genitals were all regarded as chlamydial symptoms (urban; *n* = 4). This suggests that in both urban and semi-urban environments, Chlamydia is the leading environmental threat.

During our study period at the AKWH, 17 koalas were diagnosed with *C. percorum* (no PCR, PCR +ve and PCR –ve). Chlamydial infections were higher in females (*n* = 12) compared to males (*n* = 5). In female infections (*n* = 12), four resulted in a final outcome of euthanasia. Gonzalez-Astudillo et al. (35) found koala females to be at a higher risk of poor clinical outcomes when diagnosed with chlamydiosis. Females have been found to express more explicit signs of chlamydiosis and the disease often causes female infertility, resulting in higher euthanasia rates in clinical settings (35). Chlamydia has been recognized as a contributing factor to koala population declines due to high incidence, detrimental impacts of the disease and the symptoms involved (35).

Clinical interventions are crucial for the appropriate care and recuperation of rescued wild koalas. Increased handling during treatment as well as a decreased success in antibiotic treatment may influence stress levels (7). Other diagnosis, such as renal failure can often be indicative of oxalate nephrosis in koalas which can be a detrimental disease to koala populations (36).

In conclusion, it is evident from the outcomes of this research that the nature of environmental stressor (trauma and/or disease) and habitat-specific demographics (location of rescue) can have influence on the physiological stress responses of wild koalas and their eventual recovery in clinical care. It is therefore important to monitor the physiological stress responses of wild rescues koalas using non-invasive techniques such as fecal glucocorticoid metabolite enzyme-immunoassays to provide early index of stress levels in koala patients and apply the data to understand how koalas perceive environmental stress (37) and improve their responses to clinical care and management.

## Author Contributions

EN conceptualized this research and collaborated with the Adelaide Koala and Wildlife Hospital. EN supervised TV for an Honors research project. TV carried out part of the lab work under the supervision of EN. EN conducted the data analysis and interpretation.

### Conflict of Interest Statement

The authors declare that the research was conducted in the absence of any commercial or financial relationships that could be construed as a potential conflict of interest.
